# Transcriptional Response of Selenopolypeptide Genes and Selenocysteine Biosynthesis Machinery Genes in *Escherichia coli* during Selenite Reduction

**DOI:** 10.1155/2014/394835

**Published:** 2014-04-15

**Authors:** Antonia Y. Tetteh, Katherine H. Sun, Chiu-Yueh Hung, Farooqahmed S. Kittur, Gordon C. Ibeanu, Daniel Williams, Jiahua Xie

**Affiliations:** ^1^Department of Pharmaceutical Sciences, Biomanufacturing Research Institute & Technology Enterprise, North Carolina Central University, Durham, NC 27707, USA; ^2^Department of Biochemistry, Kwame Nkrumah University of Science and Technology, Kumasi, Ghana; ^3^School of Geography and Geosciences, University of St Andrews, St Andrews, Fife KY16 9AX, UK; ^4^Department of Biology, North Carolina Central University, Durham, NC 27707, USA

## Abstract

Bacteria can reduce toxic selenite into less toxic, elemental selenium (Se^0^), but the mechanism on how bacterial cells reduce selenite at molecular level is still not clear. We used *Escherichia coli* strain K12, a common bacterial strain, as a model to study its growth response to sodium selenite (Na_2_SeO_3_) treatment and then used quantitative real-time PCR (qRT-PCR) to quantify transcript levels of three *E. coli* selenopolypeptide genes and a set of machinery genes for selenocysteine (SeCys) biosynthesis and incorporation into polypeptides, whose involvements in the selenite reduction are largely unknown. We determined that 5 mM Na_2_SeO_3_ treatment inhibited growth by **∼**50% while 0.001 to 0.01 mM treatments stimulated cell growth by **∼**30%. Under 50% inhibitory or 30% stimulatory Na_2_SeO_3_ concentration, selenopolypeptide genes (*fdnG*, *fdoG*, and *fdhF*) whose products require SeCys but not SeCys biosynthesis machinery genes were found to be induced ≥2-fold. In addition, one sulfur (S) metabolic gene *iscS* and two previously reported selenite-responsive genes *sodA* and *gutS* were also induced ≥2-fold under 50% inhibitory concentration. Our findings provide insight about the detoxification of selenite in *E. coli* via induction of these genes involved in the selenite reduction process.

## 1. Introduction

Selenium (Se) is a nonmetal element with atomic number 34, which is chemically related to sulfur (S) and tellurium but rarely found in its elemental form in nature. Se is an essential micronutrient for mammals and some bacteria and a component of selenocysteine (SeCys), an amino acid used by a group of proteins [1, 2]. In* Escherichia coli* genome, there are three formate dehydrogenases FdhN, FdhO, and FdhH with each consisting of one selenopolypeptide requiring SeCys [3–6] for the oxidation of formate and carbon dioxide [2]. At elevated concentrations, however, Se can be toxic [7, 8]. High Se levels are known to produce reactive oxygen species that cause DNA damage and various diseases in mammals [8, 9]. Previous studies have shown that some bacteria, unlike mammals and yeasts, can tolerate high levels of Se through the reduction of toxic selenate and selenite to an insoluble, less toxic, red Se element (Se^0^) [10–13]. In nature, selenate and selenite are two major types of soluble inorganic compounds. Selenite is more toxic than selenate and other forms of Se compounds [14]. Hence, the reduction of selenite by microorganisms has a broad biological importance.

Several bacterial species including* E. coli* have the capacity to convert selenite to Se^0^ [15–27].* E. coli* strains can grow in the presence of 9.2 mM selenite and efficiently metabolize selenite into Se^0^ [12]. However, the detailed processes of how selenite is reduced, what molecular mechanism is utilized, and how bacterial cells respond to selenite are still unknown [13, 28] although the physiological mechanisms of selenite reduction have been studied in several species [12, 15, 26]. It is believed that selenite like selenate may enter the cells through the sulfate permease system controlled by* cysA*,* cysU*, and* cysW* [12]. Selenite may also enter the cells through an alternative system, such as the sulfate transporter, because disruption of sulfate permease expression did not completely block its uptake [12]. After entry into the cells, selenite may be reduced to selenide [12], followed by oxidation to Se^0^ [29]. Further study on the molecular mechanism involved in selenite reduction is necessary, which will assist in understanding the detoxification of selenite.

Currently, the knowledge concerning the molecular mechanism involved in selenite reduction in* E. coli* is limited to four genes that have been identified to respond to sodium selenite (Na_2_SeO_3_) treatment. Three of them are oxidative stress stimulons* sodA*,* gor*, and* trxB*, encoding the manganese superoxide dismutase, glutathione reductase, and thioredoxin reductase, respectively, which are upregulated ≥5-fold by 2 mM Na_2_SeO_3_ treatment [30]. The fourth gene is* gutS* (or called* yhfC*) that encodes the GutS polypeptide, a homolog of membrane transport proteins, which can be induced by 0.03–0.06 mM Na_2_SeO_3_ and 0.002 mM sodium tellurite [31]. Microarray analysis was employed to investigate transcript changes genome-wide in* Caulobacter crescentus* cells treated with 0.3 mM Na_2_SeO_3_; only 12 genes were found to be upregulated about 2- to 5-fold [32]. However, all of these genes were also induced by chromium and cadmium [32], suggesting that they are more likely to be general stress induced genes rather than specifically responding to selenite. No homologous genes of the aforementioned four selenite-induced genes involved in oxidative stress [30] and membrane transport [31] were found to be upregulated by Na_2_SeO_3_ in* C. crescentus* [32]. Therefore, in depth analysis is essential to understand whether any additional genes in bacterial genome are responsible for selenite reduction.

In* E. coli*, there are only three proteins FdhN, FdhO, and FdhH that each contains one polypeptide requiring SeCys residue for their activities [3–6]. The gene* fdnG* encodes a 110 kD selenopolypeptide, *α* subunit of FdhN. The* fdoG* encodes another 110 kD selenopolypeptide for FdhO while the* fdhF* encodes a 80 kD selenopolypeptide of FdhH. In addition, there is a group of SeCys biosynthesis and incorporation machinery genes for the biosynthesis of SeCys and selenopolypeptides such as* selA* (selenocysteine synthase),* selB* (selenocysteinyl-tRNA-specific translation factor),* selC* (tRNA specific for selenocysteine),* selD* (selenophosphate synthase) [6, 33],* ybbB* (selenophosphate-dependent tRNA 2-selenouridine synthase) [34], and* sufS* (PLP-dependent selenocysteine lyase) [35]. We questioned how these genes respond during selenite reduction. To evaluate the changes in transcript levels of Se metabolic genes, Na_2_SeO_3_ concentrations that are 30% stimulatory and 50% inhibitory on bacterial cell growth were determined and used to study their expressions by quantitative real-time PCR (qRT-PCR). In addition, microarray analysis was also used to screen selenite-responsive genes besides above selected genes.

## 2. Materials and Methods 

### 2.1. Bacterial Strain and Growth Conditions

*E. coli* strain K12 was used in this study. Bacterial cells were grown in Luria-Bertani (LB) medium in 14-mL polystyrene round-bottom tubes. A final volume of 3 mL was used for cultures and incubated at 37°C in a rotary shaker at 225 rpm for different periods of time depending on the experiment.

### 2.2. Screening Inhibitory and Stimulatory Concentrations

To determine which concentrations of Na_2_SeO_3_ inhibited and stimulated bacterial growth, an overnight culture of K12 adjusted to the optical density at 600 nm (OD_600_) of 1 was used as bacterial stock solution for testing. Na_2_SeO_3_ (Sigma-Aldrich) was dissolved in ddH_2_O to prepare 1 M selenite stock solution. Initially, each 20 *μ*L of bacterial stock solution was inoculated into 3 mL LB medium containing Na_2_SeO_3_ with a final concentration of 0, 25, 50, 75, 100, or 125 mM. The experiment was performed on three biological replicates in triplicate for each concentration tested. After 14 h of growth, the extent of selenite to Se^0^ reduction as indicated by the degree of red color in each culture was determined qualitatively and photographs were compared. Since reduced red Se^0^ can interfere with optical density measurements, bacterial growth was determined by counting viable cell numbers. Cells were diluted using LB medium and then 20 *μ*L of diluted culture was spread onto a LB agar and incubated at 37°C overnight. The serial dilutions were done in triplicate for each culture. Thus, the colony-forming units (CFUs) were calculated from nine plates for each treatment.

After the initial test, 0, 5, 10, 15, 20, 25, and 30 mM Na_2_SeO_3_ were used to determine the concentration that would inhibit the bacterial growth. Another experiment using lower concentrations (0, 0.0001, 0.001, 0.01, 0.05, 0.1, 0.5, 1, 5, and 10 mM) was performed to determine which concentration stimulated bacterial growth. Both experiments were performed on three biological replicates in triplicate for each concentration tested.

### 2.3. Determining a Time Point of  Significant Growth Difference between 5 mM Na_2_SeO_3_ Treated and Untreated Cultures

After screening the inhibitory selenite concentration, cultures were treated with selected 5 mM Na_2_SeO_3_ to determine when cells should be harvested for RNA extraction at which significant difference in bacterial growth between treated and untreated control could be observed. The same culture conditions were used as previously mentioned; treated and untreated cultures were incubated at 37°C for 0, 2, 4, 6, 8, 10, 12, or 14 h. Colony forming units were recorded as previously mentioned for each time point. Three biological replicates done in triplicate were done for each culture.

### 2.4. Preparing 0.5- and 2-h Cultures for qRT-PCR Analysis

To study the effect of culture duration on induced selenite-responsive genes, two previously reported growth times, 0.5 h [30, 32] and 2 h [30, 31], and selected 6 h were tested. To determine an optimal initial culture concentration which allows cells to reach log phase of growth after 0.5 and 2 h short culture periods, cells were precultured to first reach OD_600_ of 0.2, 0.4, and 0.8; then, these were served as initial inoculant concentrations for culturing for 0.5, 1, 1.5, 2, 2.5, and 3 h. The cell density of each culture was recorded by measuring OD_600_ value. Three biological replicates with triplicate assay for each time point were done for the entire experiment.

### 2.5. RNA Isolation

Total RNA was isolated using the RiboPure-Bacteria RNA Isolation Kit (Applied Biosystems/Ambion, USA) and treated with DNase I (Applied Biosystems/Ambion, USA) to remove any DNA contamination. RNA concentration was quantified using a NanoDrop ND 1000 spectrophotometer. The quality of the RNA was visualized on a 1.2% agarose gel. For microarray analysis, RNA quality was further checked using a dual beam spectrophotometer and an Agilent Bioanalyzer 2100 Lab-on-a-Chip system.

### 2.6. qRT-PCR Analysis

Three selenopolypeptide genes (*fdnG*,* fdoG*, and* fdhF*) and five machinery genes (*selA*,* selB*,* selD*,* ybbE*, and* sufS*) for SeCys biosynthesis and insertion were selected to quantify their expression levels in response to selenite treatments. The* selC* was not selected because its sequence (95 bp) is too short to meet the requirement of primer design software Primer Express 3.0 (Applied Biosystems/Ambion, USA) for designing a pair of primers. Previously reported four selenite-induced genes (*gor*,* sodA*,* trxB*, and* gutS*) [30, 31] and three S metabolism related genes (*sbp*, sulfate transporter subunit,* thiP*, thiamine transporter membrane protein, and* iscS*, cysteine desulfurase) [36–38] were included for comparison. Bacterial cells untreated and treated either with 0.01 or 5 mM Na_2_SeO_3_ were used for RNA isolation. Equal amounts of RNA for each sample were used to synthesize first-strand of cDNA with a High Capacity cDNA Reverse Transcription kit and random primers (Applied Biosystems, USA). PCR was carried out with the Power SYBR Green mix (Applied Biosystems, USA). Primer design and ΔΔCt calculation were carried out as described previously [39]. Sequences of selected genes were retrieved from strain K12 genome (accession no. AC_000091) deposited in NCBI (http://www.ncbi.nlm.nih.gov/) for designing primers. The* 16S rRNA* was used as an endogenous control.* 16S rRNA* primers were designed based on 719 bp conserved region of seven members (*rrsA*,* B*,* C*,* D*,* E*,* G*, and* H*) also retrieved from strain K12 genome. Each sample was assayed in triplicate and the experiment was repeated with three biological replicates. Detailed primer information for each gene is listed in Table 1.

### 2.7. Microarray Analysis

Bacterial cells untreated and treated with 0, 0.01, or 5 mM Na_2_SeO_3_ for 6 h were used for microarray analysis for screening additional selenite-responsive genes which were not in the previous qRT-PCR analysis. Three replicates for treated and untreated samples were used. The microarray chip used in this experiment was the Affymetrix GeneChip* E. coli* genome 2.0 arrays (Affymetrix, USA) containing 10,208 probe sets for detecting the entire 20,366 genes in the K12 strain and three other* E. coli* strains. The RNA quality control analysis, standardization, cRNA labeling, and array hybridization were processed at Genome Explorations Inc. (http://www.genome-explorations.com/, USA). Raw signals (CEL files) were normalized and transformed into log_2_ values by MAS 5 (scaled to TGT = 250). For statistical analysis, the significance analysis tool set in gene traffic was employed to perform multiclass ANOVA. Pairwise comparisons were made between untreated control and 0.01 or 5 mM treatment. All probe sets having comparisons reaching absolute fold change ≥1.5 and *t*-test *P* values ≤0.05 were selected for further qRT-PCR confirmation. Aforementioned qRT-PCR conditions and endogenous control were used. Sequences of related genes were retrieved from strain K12 genome. Detailed primer information for each gene is listed in Table 1.

### 2.8. Statistical Analysis

For the comparison of cell numbers between untreated control and Na_2_SeO_3_ treatments, Student's *t*-test was used.

## 3. Results

### 3.1. Na_2_SeO_3_ Concentration Causing 50% Inhibitory Effects

**Figure 1:**
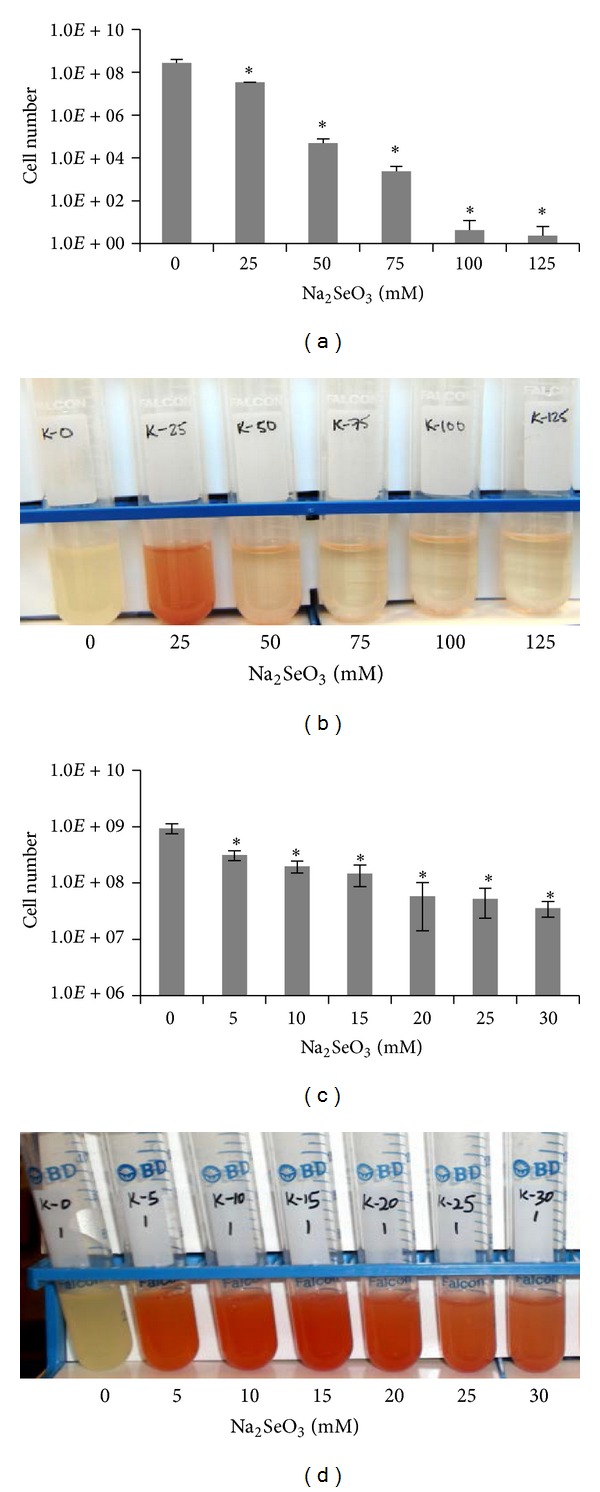
Effect of Na_2_SeO_3_ treatment on* E. coli* cell growth and selenite reduction. The number of bacterial cells under 0 to 125 mM Na_2_SeO_3_ treatments (a) and 0 to 30 mM Na_2_SeO_3_ treatments (c). The experiment was performed with three biological replicates and each Na_2_SeO_3_ treated sample was analyzed in triplicate. Data plotted was the average of cell numbers ± SD. A representative of change in color of cultures treated with 0 to 125 mM Na_2_SeO_3_ (b) and 0 to 30 mM Na_2_SeO_3_ (d). *P ≤ 0.05.

To examine Na_2_SeO_3_ inhibitory effects on bacterial growth,* E. coli* K12 was cultivated in the presence of high concentration of Na_2_SeO_3_ at 0, 25, 50, 75, 100, or 125 mM. Results showed that all these selenite treatments caused more than 50% inhibition of* E. coli* growth (Figure 1(a)). Overnight treatment using 25 mM selenite inhibited bacterial growth about 8-fold compared to untreated cultures (Figure 1(a)) and led the culture to turn red (Figure 1(b)), indicating that selenite reduction has occurred. When 50 mM or higher selenite concentrations were used, cell numbers drastically decreased and these cultures turned slightly but not completely red. This suggests that selenite reduction was decreased under high selenite concentrations, which could be due to extremely low number of bacterial cells.

A narrower range of Na_2_SeO_3_ (0 to 30 mM with a 5 mM interval) was used to determine a concentration of Na_2_SeO_3_ that would inhibit bacterial growth by 50%. We found that 5 mM Na_2_SeO_3_ treatment reduced cell numbers by 53% and turned cultures red (Figures 1(c) and 1(d)). When concentrations of 10 to 30 mM were used, cell numbers were reduced by 79–97% and all these cultures still turned red, especially at 10 and 15 mM. These results are consistent with the previous observations that selenite can be reduced to red Se^0^ by* E. coli* [12, 30] and indicate that the selenite reduction occurred efficiently in* E. coli* K12 cells treated with 5 to 30 mM Na_2_SeO_3_.

### 3.2. Na_2_SeO_3_ Concentration Having Stimulatory Effects

**Figure 2:**
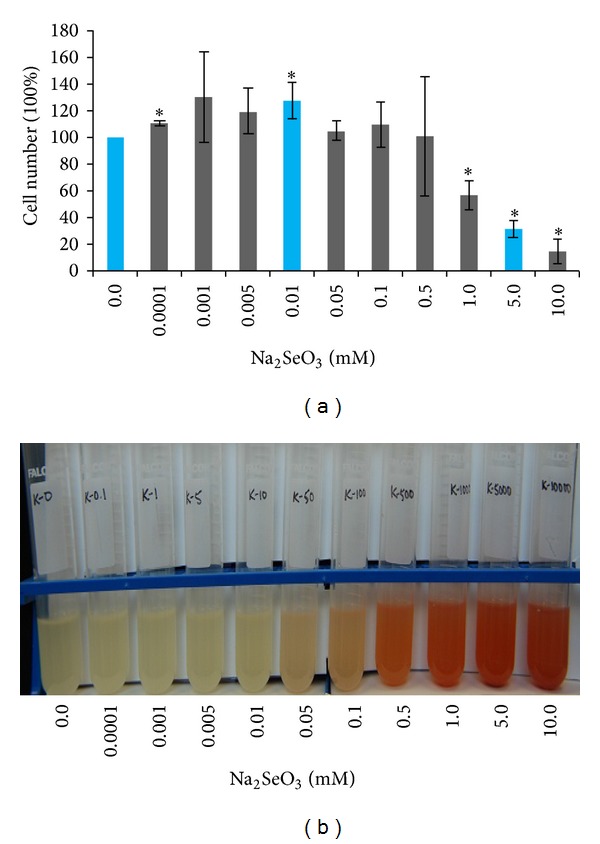
Effect of low concentrations of Na_2_SeO_3_ treatment on* E. coli* cell growth and selenite reduction. (a) The effect of 0 to 10 mM Na_2_SeO_3_ on cell growth rates. The experiment was performed with three biological replicates and each Na_2_SeO_3_ treated sample was analyzed in triplicate. Data plotted was the average of growth ± SD. The concentrations (0, 0.01, and 5 mM) that were marked in blue were used to prepare RNA samples for microarray analysis. (b) Color change in cultures after treatments with 0 to 10 mM Na_2_SeO_3_. *P ≤ 0.05.

Se is an essential element for bacterial growth. To determine what Na_2_SeO_3_ concentration range stimulating bacterial growth, 0 to 10 mM Na_2_SeO_3_ was tested. Compared to the untreated control, 0.0001 to 0.1 mM Na_2_SeO_3_ treatments could stimulate K12 cell growth (Figure 2(a)). The stimulatory effects were increased from 10% to 30% when the concentrations were increased from 0.0001 to 0.001 mM. However, no significant color changes were observed in 0.0001 to 0.01 mM treated cultures, indicating no or very low levels of selenite reduction occurring (Figure 2(b)). The stimulatory effects of selenite on the cell growth were decreased with increasing in Na_2_SeO_3_ concentrations (Figure 2(a)). When 0.05 to 0.5 mM treatments were used, the cultures turned red (Figure 2(b)) but the stimulatory effects of these treatments on bacterial growth were slight (Figure 2(a)). Consistently, more than 50% inhibitory effects with clear red color in cultures were observed in this experiment when Na_2_SeO_3_ concentrations reached as high as 5 and 10 mM. Based on the above results, 0.01 mM was selected as a stimulatory concentration for further studies.

### 3.3. Expression Levels of Selected Genes under Inhibitory and Stimulatory Conditions

**Figure 3:**
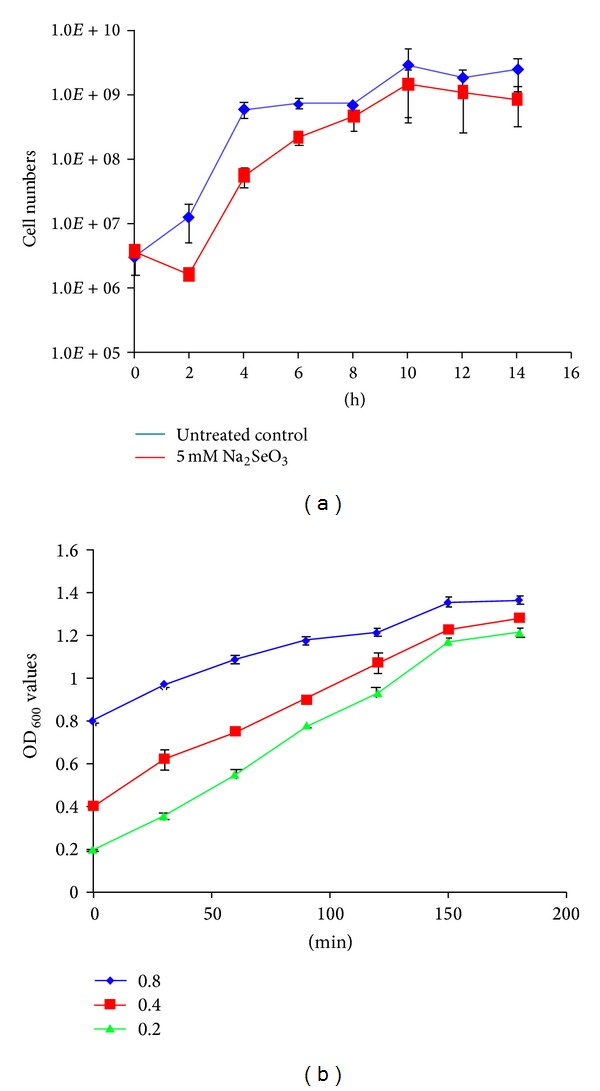
The growth curve of bacterial cells. The growth curve of cells cultured with or without 5 mM Na_2_SeO_3_ (a). The growth curve of cells cultured with initial culture concentrations of OD_600_ values of 0.2, 0.4, and 0.8 (b). Each experiment was performed with three biological replicates and each treatment was assayed in triplicate. Data plotted was the average ± SD.

To investigate how selenopolypeptide genes and machinery genes for SeCys biosynthesis and insertion respond to Na_2_SeO_3_ concentrations that cause 30% stimulatory or 50% inhibitory to bacterial cell growth, their transcript levels were quantified by qRT-PCR. For comparison, harvesting Na_2_SeO_3_-treated and untreated cultures at a time point where the most significant difference in growth is important. To this end, a ~50% inhibitory concentration of 5 mM Na_2_SeO_3_ was used for cultures and bacterial growth was monitored every 2 h for 14 h. Cell densities were significantly different between treated and untreated cultures after being grown for 2 and 4 h (Figure 3(a)). The number of cells in treated culture was reduced during the first 2 h of growth, which suggests that some cells may have died during the initial culture stage. Those survival cells gradually caught up the growth and reached the highest cell numbers at 10 h. The untreated cultures, on the other hand, reached stationary phase early at 6 h, but the highest cell numbers were also at 10 h. Therefore, 6 h was selected as a time point to harvest cells for quantifying gene expression levels.

To investigate the expression of selenite-responsive genes at different culture time point, previously reported growth times of 0.5 [30, 32] and 2 h [30, 31] were also used as well as 6 h treatment. However, the number of cells could be too low to isolate a usable quality and quantity of RNA after growing for 0.5 and 2 h if the same 20 *μ*L bacterial stock solution (OD_600_ = 1) is inoculated into 3 mL LB medium. To determine which initial culture concentration would allow the culture to reach log phase after culturing for 0.5 to 3 h, a pretest culture experiment was performed with initial OD_600_ of 0.2, 0.4, and 0.8. The results showed that OD_600_ values increased from initial 0.2, 0.4, and 0.8 to 0.4, 0.6, and 0.9 after culturing for 0.5 h and to 0.9, 1.1, and 1.2 after culturing for 2 h, respectively (Figure 3(b)). Based on these observations, bacterial growths reached to log phase after 0.5 and 2 h cultures when the initial OD_600_ of 0.4 was used. Therefore, cultures with initial OD_600_ of 0.4 treated with 0.01 or 5 mM Na_2_SeO_3_ for either 0.5 or 2 h were used to study gene expression.

**Figure 4:**
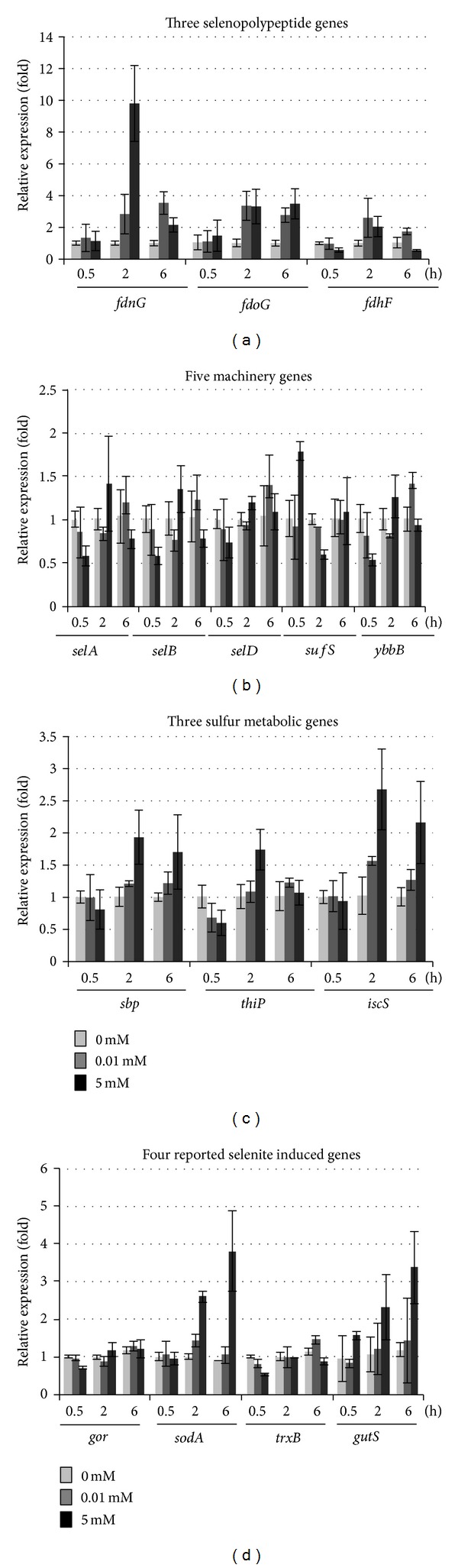
Transcript levels of selected genes quantified by qRT-PCR. (a) Three selenopolypeptide genes:* fdnG*,* fdoG,* and* fdhF*. (b) Five machinery genes for SeCys biosynthesis and insertion:* selA*,* selB*,* selD*,* ybbE*, and* sufS*. (c) Three S metabolic genes:* sbp*,* thiP*, and* iscS*. (d) Four previously reported selenite-induced genes:* gor*,* sodA*,* trxB*, and* gutS*. The RNA samples were isolated from bacteria cells grown under 0, 0.01, or 5 mM Na_2_SeO_3_ for 0.5, 2, or 6 h. Data shown are fold changes calculated as transcript levels of selenite treated samples compared to untreated (defined as 1). Data represent an average of three biological replicates ± SD. Each replicate was assayed in triplicate.

QRT-PCR results showed that two selenopolypeptide genes* fdnG* and* fdoG* were induced 2- to 10-fold by 0.01 or 5 mM Na_2_SeO_3_ with 2 or 6 h treatments, but not at 0.5 h (Figure 4(a)). The third one* fdhF* was induced about 2-fold by 0.01 and 5 mM Na_2_SeO_3_ treatments for 2 h only. The expression levels of all five SeCys biosynthesis and insertion machinery genes* selA*,* selB*,* selD*,* ybbE*, and* sufS* did not show ≥2-fold changes in any of the treatments (Figure 4(b)). These results suggest that maintaining the constant expression of these machinery genes is necessary for survival. Concerning three S metabolic genes, only* iscS* was induced 2.7- and 2.2-fold in 5 mM treatment for 2 and 6 h (Figure 4(c)). The remaining two genes in all treatments and* iscS* in remaining treatments did not cause ≥2-fold changes in their expression levels. When four previously reported selenite induced genes were analyzed, two of them (*sodA* and* gutS*) were induced 2.6- and 3.8-fold in 5 mM treatment for 2 and 6 h but not in the other treatments (Figure 4(d)). The expression levels of the other two genes (*gor* and* trxB*) did not meet the 2-fold cutoff under either 0.01 or 5 mM treatment, which is not in agreement with previous report by Bébien et al. [30].

### 3.4. Identification of Additional Selenite-Responsive Genes by Microarray Analysis

Microarray analysis is a powerful approach to study differential gene expression by genome-wide screening simultaneously. In order to identify genes besides above selected genes that are responsive to current selenite treatment conditions, microarray analysis was performed to compare transcript levels of genes in whole genome between untreated cells and 0.01 or 5 mM and 6 h treated cells. RNA samples from three biological replicates per treatment were used for hybridization. The results showed that average hybridization signals of probe sets were 554.4, 557.0, and 560.1 while background signals were 39.7, 45.8, and 47.4 for untreated, 0.01 mM treated, and 5 mM treated samples, respectively (Table 2). These results indicate that hybridization and scanning were efficient. After pairwise comparison of data using statistical analysis, fold changes of all genes between the untreated and 0.01 or 5 mM treated were calculated. Genome-wide comparison with 4326 genes in the K12 genome [40] by microarray revealed that nearly no single gene had its transcript level change more than 2-fold when cultures were grown under either 30% stimulatory or 50% inhibitory selenite conditions (data not shown). It was surprising to note that transcript levels of all above selenite-responsive genes detected by qRT-PCR did not display difference between treated and control. Only expression levels of* yegB* (probe set ID 1764732) were induced 2.2- and 1.8-fold by 0.01 and 5 mM treatments, respectively (Table 2), but its average hybridization signal in control group was 27.0, which is below the background signal 39.7 (Table 2).

**Figure 5:**
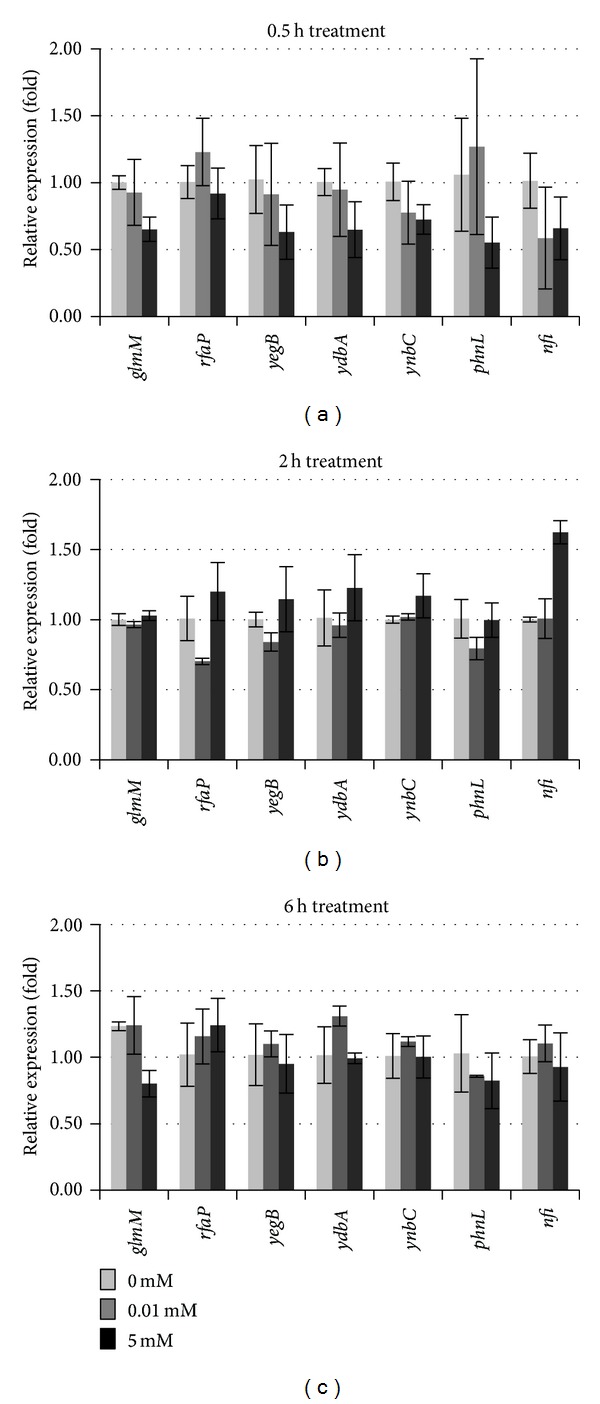
Transcript levels of seven selenite-responsive candidate genes quantified by qRT-PCR. The RNA samples were isolated from bacteria cells grown under 0, 0.01, or 5 mM Na_2_SeO_3_ treatment for (a) 0.5, (b) 2, or (c) 6 h. Data shown are fold changes calculated as transcript levels of selenite treated samples compared to untreated (defined as 1). Data represent an average of three biological replicates ± SD. Each replicate was assayed in triplicate.

Using absolute fold change ≥1.5 and *P* values ≤0.05 as a standard, three genes in K12 genome were induced in their expressions while two genes were inhibited by 0.01 mM Na_2_SeO_3_ treatment for 6 h (Table 2). When K12 cells were treated with 5 mM for 6 h, only two genes were induced while one was inhibited in their expressions. Among these identified selenite-responsive candidate genes, the* yegB* was induced ≥1.5 by both 0.01 and 5 mM treatments. However, all these candidate genes had relatively low hybridization signals (Table 2). When qRT-PCR was used to confirm expression levels of these selenite-responsive candidate genes in cells treated with 0.01 or 5 mM selenite for 0.5, 2, or 6 h, none of them had their expression level changed more than 1.5-fold under either stimulatory or inhibitory conditions (Figure 5).

## 4. Discussion

Previous studies have shown that* E. coli* cells exhibit three types of responses when subjected to selenite treatment. At extremely low concentrations (<0.0002 mM), selenite is readily incorporated into SeCys for the synthesis of selenoproteins, such as formate dehydrogenases [4]. At moderate concentrations (>0.001 mM), selenite intrudes the S metabolic pathway and is metabolized along the routes of  S metabolism, but never affects cell growth until it reaches 0.08 mM [41, 42]. At higher concentrations (>5 mM), selenite becomes toxic* via* the mechanism of oxidative stress [30]. In the present study, 0.001 to 0.01 mM sodium selenite concentrations were found to promote bacterial growth approximately by 30% (Figure 2(a)). The trend of all four concentrations used for stimulatory effect was the same although 0.001 and 0.05 mM treatments did not have significantly stimulatory effects. Meanwhile, 5 mM treatment inhibited cell growth steadily by more than 50% (Figures 1(c), 2(a), and 3(a)). The results of our growth studies are consistent with previous reports [4, 30, 41, 42] which lay a foundation for further investigating selenite-responsive genes.

Using qRT-PCR analysis, we found that all three selenopolypeptide genes* fdnG*,* fdoG*, and* fdhF* encoding selenopolypeptides for FdhN, FdhO, and FdhH were induced more than 2-fold by both 0.01 and 5 mM Na_2_SeO_3_ (Figure 4(a)), which have not been reported previously.* E. coli* has only these three proteins with each containing one selenopolypeptide requiring SeCys for its activity [3–6]. A possible role for SeCys in these enzymes could be important for the adjustment of the redox potential, making catalytic reaction possible without oxygen transfer [43]. FdhN and FdhO are known to be responsible for the oxidation of formate and transfer of electrons from formate to nitrate [44, 45]. Therefore, observed results could be simply explained that induced expression of these genes encoding selenopolypeptides by selenite is only for the protection of bacterial cells via their antioxidation functions. This could be reasonable if* fdnG*,* fdoG*, and* fdhF* are only induced by 5 mM Na_2_SeO_3_ treatment. Since they were induced more than 2-fold by 0.01 mM Na_2_SeO_3_ as well, whether these proteins containing SeCys have an additional function other than antioxidation needs to be further investigated. In the presence of 0.01 mM Na_2_SeO_3_, a small part of selenite may be used to synthesize selenopolypeptides while large part may be metabolized with the S as suggested by previous studies [41, 42]. Nevertheless, these results suggest that these selenopolypeptide genes are either directly or indirectly involved in selenite reduction.

Moreover, we also found that one sulfur metabolic gene* iscS* and two previously reported selenite-induced genes* sodA* and* gutS* were induced only by 5 mM (Figure 4(d)), which indicate these genes are also involved in selenite reduction. Under 5 mM Na_2_SeO_3_ treatment, bacterial cells were already under toxic condition with more than 50% growth inhibition even though the selenite reduction was still active as judged from the accumulation of Se^0^ (Figures 1 and 2). It is known that under the toxic conditions, the reduction of selenite involves reactions with sulfhydryl groups of thiol-containing molecules, such as glutathione, and some of these reactions produce reactive oxygen species: hydrogen peroxide and superoxide [12, 30]. Both hydrogen peroxide and superoxide can cause damage to cell membranes and DNA [46]. Therefore, the gene like* sodA* encoding the antioxidant enzyme [30] is induced in response to oxidative stress. However, it is not clear why previously reported selenite inducible genes* gor* and* trxB*, encoding antioxidant proteins glutathione reductase and thioredoxin reductase, respectively [30], could not be induced by selenite treatment in the present study. Induced expression of* gutS* by selenite was similar to the previous report [31]. The* gutS* gene product may allow Se to permeate into cells because it shares homology with membrane transport proteins. Induction of* iscS* under the selenite toxic conditions (Figure 4(c)) may be due to its dual function in both S and Se metabolisms. Unlike the other two sulfur metabolic genes, the* iscS* is also required for biosynthesis of 2-selenouridine in tRNA and FdhH [47].

In the present study, we also made an attempt to identify selenite-responsive genes genome-wide by microarray screening. Surprisingly, this screening did not yield any selenite-responsive genes in cells treated with 50% inhibitory or 30% stimulatory Na_2_SeO_3_ concentration. Our results of microarray experiment are similar to those reported by Hu and coworkers [32], in which only a few general stress-induced genes were identified in their microarray screening. Although underestimation of the fold changes of differentially expressed genes in a microarray assay was reported previously in comparison with the qRT-PCR analysis [48], Hu and coworkers (2005) [32] could still use microarray analysis to identify chromium and cadmium (but not Se) induced genes in bacterium. Our current microarray results in conjunction with microarray screening results reported by Hu et al. (2005) [32] from selenite treated bacterial cells suggest that selenite reduction may result from minor contributions from a small set of genes with slight changes at transcript levels and that microarray analysis may not be sensitive enough to identify those selenite-responsive genes, which could be identified by qRT-PCR.

## 5. Conclusions

We have comprehensively studied the bacterial growth under various concentrations of Na_2_SeO_3_ and determined that 0.001 to 0.01 mM promoted bacterial growth approximately by 30%, whereas 5 mM treatment inhibited cell growth by more than 50%. Although microarray analysis was not sensitive enough to identify these selenite-responsive genes in bacteria, we were able to determine that genes encoding selenopolypeptides and some antioxidant proteins were involved in selenite reduction. Our findings will help to further elucidate the mechanism responsible for selenite reduction.

## Figures and Tables

**Table 1:** Primers used for qRT-PCR.

Gene name	Forward primer	Reverse primer
*fdnG *	5′-CCGAAGTGGGACCAGACCTA-3′	5′-TGACTTTGCCTTCATCCATCAT-3′
*fdoG *	5′-GCAGATCCGCAGGGTAACC-3′	5′-CTTAGTGCCGTCCCATTTCAG-3′
*fdhF *	5′-TTCTGTACGTGAAGCGACGAA-3′	5′-GCGATGGGCCATGTCATTAT-3′
*selA *	5′-CCGAAACGCGTTCCCTCTA-3′	5′-GGAGCTATCGCGCAATAAGC-3′
*selB *	5′-TCGCGTGCCTGGTTTTATC-3′	5′-GCCAGCATGTTGGAAAGAAACT-3′
*selD *	5′-ATAACGCTGGTGCCATTGC-3′	5′-GTAATGAAACCCGCGACGAT-3′
*sufS *	5′-CCGCGTAGCCATGACGATA-3′	5′-CAGAAACCGAGCCAGGTGAT-3′
*ybbB *	5′-CGTACGCGTGGGTAAAATCA-3′	5′-TGAGCGCCTGAACGAAGAGT-3′
*sbp *	5′-CCACCGTGTGACTGACGAAT-3′	5′-AGCGCCCACTGGAAACAG-3′
*thiP *	5′-AGGGTGCGGAAATCATCGT-3′	5′-GCTGTCGATTGGTGATTTTGG-3′
*iscsS *	5′-GGCCGGGTTACCAAAGGTT-3′	5′-GCGTGTTGCCGAGAAAATG-3′
*gor *	5′-ATCGGAAGAGAAGATTGTCGGTAT-3′	5′-CCCTGCAACATTTCGTCCAT-3′
*sodA *	5′-TCTCCGCTGATGGGTGAAG-3′	5′-CACATCCAGGCCCATAATCG-3′
*trxB *	5′-TGCCGGTCTGTTTGTTGCT-3′	5′-GCCCTTCGAAAATCGCAGTA-3′
*gutS *	5′-TGGAAATCGTCCCGTTGAA-3′	5′-GCCAGCACCATCAGGAGAAA-3′

*16S *	5′-TTTACGCCCAGTAATTCCGATT-3′	5′-CCAGCAGCCGCGGTAATA-3′

*glmM *	5′-AGCGGCGATCCACTTGAG-3′	5′-CCAGCGCAGCTTCAACCT-3′
*rfaP *	5′-GCGGATGCCCGTTTTG-3′	5′-CATCACTCAGGCGATGAATAGC-3′
*yegB *	5′-GGTTGGCATGGCGGTATTAA-3′	5′-TCAGCGGCGATAAACCTGTA-3′
*ydbA *	5′-CGCCATATGCGGGTGTAAA-3′	5′-GCATTGCGCTCCTGATAGC-3′
*ynbC *	5′-CGTCTGCGTGGTCTGTTTTTT-3′	5′-GCCGATCGTGGGTCAAATAG-3′
*phnI *	5′-TTTTGGCTTATTGGTGGATGTG-3′	5′-TTACCGCAGAGCCGTTTTTT-3′
*nfi *	5′-CGTCGGCGAACTGCTGAT-3′	5′-CGGCGGGTCGATAAAACC-3′

**Table 2:** Hybridization signals of average of all probe sets and seven selected probe sets and fold changes of seven selenite-responsive candidate genes from microarray analysis.

Probe set ID^a^	Gene name	Signals/encoding enzymes	Microarray hybridization signals^b^			Fold changes^c^	
			0	0.01 mM	5 mM	0.01 mM	5 mM
		Average of all probe sets	554.2 ± 2.3	557.0 ± 1.9	560.2 ± 1.8		

1762328	*glmM *	Phosphoglucosamine mutase	61.0 ± 15.4	64.3 ± 6.8	32.0 ± 4.7	1.1	−1.9
1764010	*rfaP *	Lipopolysaccharide core biosynthesis protein rfaP	33.7 ± 1.7	51.8 ± 9.5	49.4 ± 1.6	1.5	1.5
1764732	*yegB *	Multidrug efflux system protein MdtE	27.0 ± 10.5	55.4 ± 3.7	48.9 ± 16	2.2	1.8
1767270	*ydbA *	Hypothetical protein	144.8 ± 17.6	90.4 ± 3.8	135.3 ± 18.2	−1.6	1.1
1768816	*ynbC *	Hypothetical protein	55.5 ± 4.6	32.0 ± 9	42.6 ± 6.1	−1.8	1.3
1768993	*phnL *	PhnI protein	50.6 ± 10.1	70.1 ± 14.2	90.7 ± 15	1.4	1.8
1768842	*nfi *	Endonuclease V	43.9 ± 6.3	72.1 ± 15	43.3 ± 11.3	1.6	1.0

^a^Selected probe sets corresponding to selenite-responsive candidate genes have absolute fold changes ≥1.5 and *t*-test *P* values ≤ 0.05 either in 0.01 or 5 mM Na_2_SeO_3_ treatment. ^b^Hybridization signals of average of all 10,208 probe sets and seven probe sets. Mean values ± SD (*n* = 3). Average of background signals are 39.7 ± 2.3, 45.8 ± 1, and 47.4 ± 0.4 for 0, 0.01 and 5 mM, respectively. ^c^Fold changes of selenite-responsive candidate genes from microarray analysis comparing to untreated control. “−” means reduced expression.
